# Supplementary material for: A Randomized Feasibility Trial of a Multicomponent Quality Improvement Strategy for Chronic Care of Cardiovascular Diseases: Findings from the C-QIP Trial in India
Source: medRxiv. 2026 Jan 30:2026.01.28.26345028. Preprint. [Version 1] doi: 10.64898/2026.01.28.26345028 (PMC12870608; doi:10.64898/2026.01.28.26345028)
Supplement: Supplement 1 [file NIHPP2026.01.28.26345028v1-supplement-1.pdf]

Supplement

Table S.1.: Fidelity of C-QIP Strategy: Self-reported cardiovascular care coordinator delivered counselling of lifestyle modifications for intervention arm participants

	Baseline (N=198)	12 months follow-up (N=114)	EOS (N=197)
**Participant counselled for lifestyle modifications**	**197 (99.5%)**	**91 (79.8%)**	**187 (94.4%)**
Healthy diet	197 (100%)	91 (100%)	186 (99.5%)
Exercise	197 (100%)	91 (100%)	184 (98.4%)
Smoking cessation *(If said yes/quit to smoking)*	24 (68.6%) (n=35)	6 (100%) (n=6)	9 (60%) (n=15)
Avoidance of stress	[illegible]	82 (90.1%)	170 (90.9%)
Abstinence/ limited use of alcohol *(If said yes to alcohol)*	10 (83.3%) (n=12)	10 (83.3%) (n=12)	2 (33.3%) (n=6)
Home monitoring of blood pressure	159 (80.7%)	83 (91.2%)	169 (90.4%)

N=number of participants

medRxiv preprint doi: https://doi.org/10.64898/2026.01.28.26345028; this version posted January 30, 2026. The copyright holder for this preprint (which was not certified by peer review) is the author/funder, who has granted medRxiv a license to display the preprint in perpetuity.
It is made available under a CC-BY 4.0 International license .

Table S.2.: Feasibility of eDSS recommended intermediate follow-up visits completion for intervention arm participants

Intermediate Visit (IV)	Expected	Completed
**Between baseline and annual visits**		
IV 2.1	195	193
IV 2.2	193	188
IV 2.3	188	180
IV 2.4	151	117
IV 2.5	97	62
IV 2.6	53	31
IV 2.7	26	13
IV 2.8	13	3
IV 2.9	3	1
**Post annual visits**		
IV 3.1	66	55
IV 3.2	41	33
IV 3.3	17	7
IV 3.4	4	1
IV 3.5	1	1

IV=Intermediate visits; eDSS=electronic decision support system

medRxiv preprint doi: https://doi.org/10.64898/2026.01.28.26345028; this version posted January 30, 2026. The copyright holder for this preprint (which was not certified by peer review) is the author/funder, who has granted medRxiv a license to display the preprint in perpetuity.
It is made available under a CC-BY 4.0 International license .

The table above shows the expected and actual number of completed intermediate visits between the baseline and annual visits, and visits completed between the first annual visit and end of the study visit.

Average follow-up visit duration between intermediate visits was approximately 3 months or as recommended per the eDSS prompts for chronic care of patients with ischemic heart disease, ischemic stroke or heart failure regardless of ejection fraction.

Figure S.1. Adoption of electronic decision support software (eDSS) prompts review and acceptance by physicians: overall and by site.

a) Antiplatelet Prompts

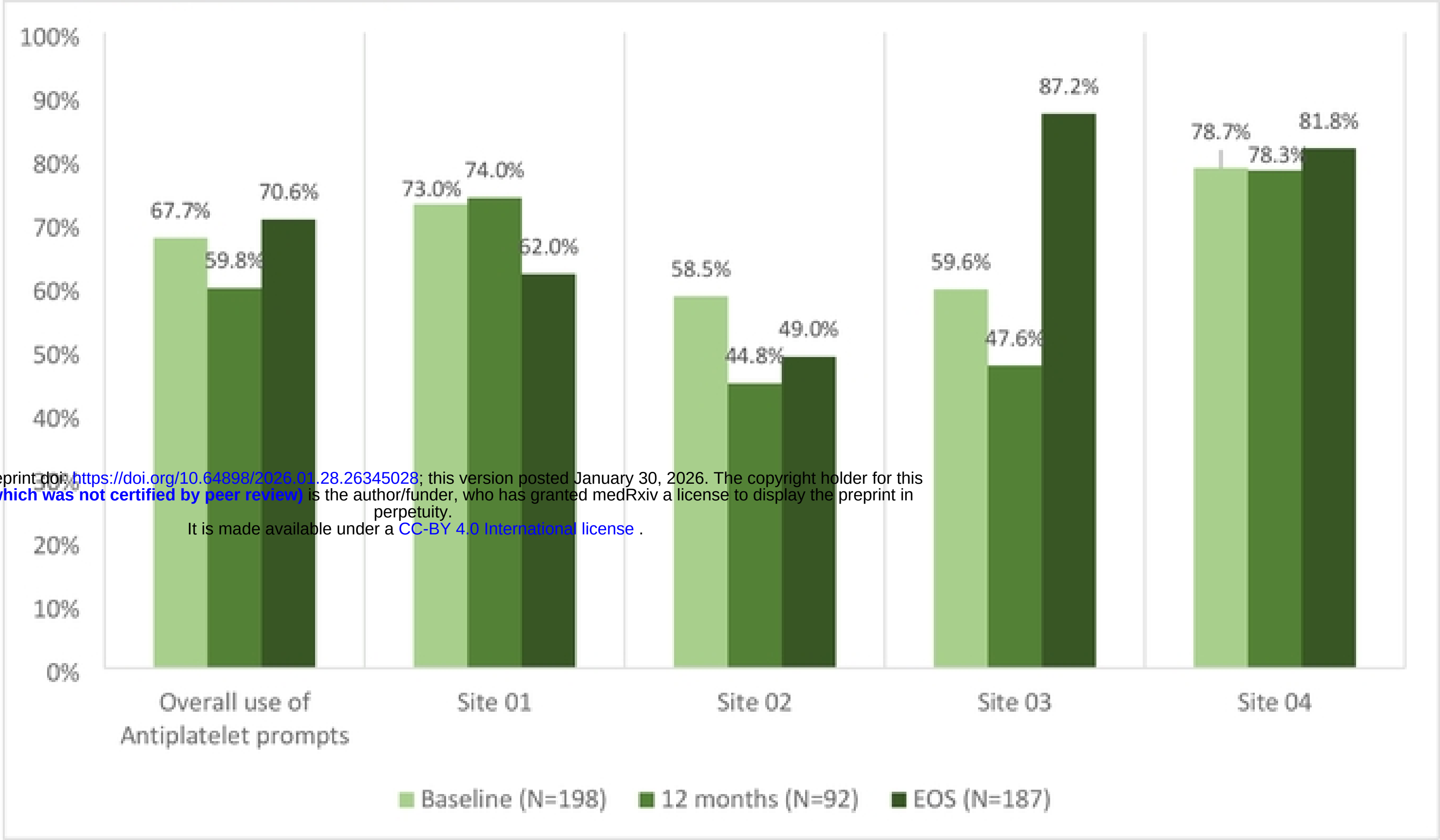

medRxiv preprint doi: https://doi.org/10.64898/2026.01.28.26345028; this version posted January 30, 2026. The copyright holder for this preprint (which was not certified by peer review) is the author/funder, who has granted medRxiv a license to display the preprint in perpetuity.
It is made available under a CC-BY 4.0 International license .

b) Blood Pressure Prompts

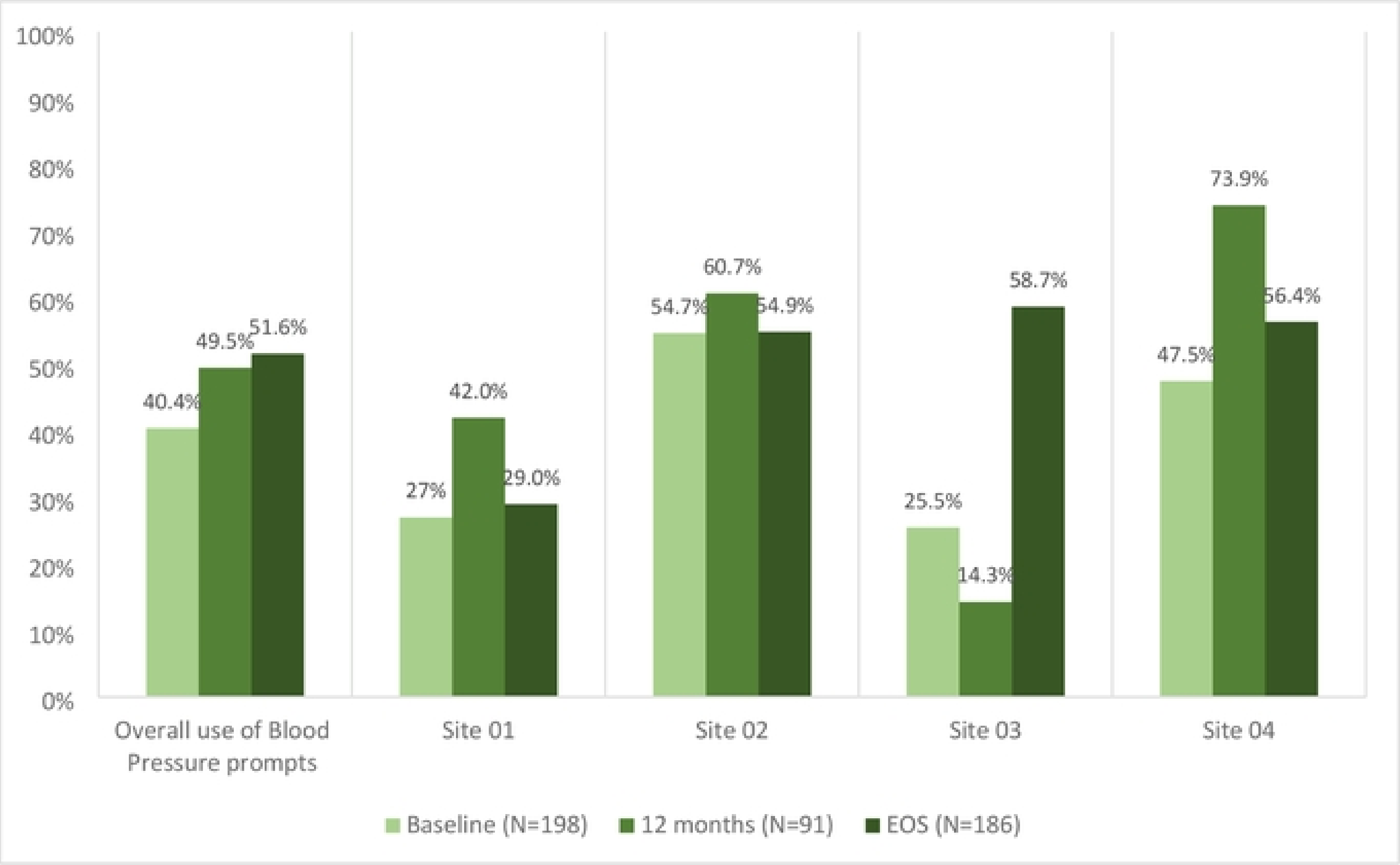

c) Lipids Prompts

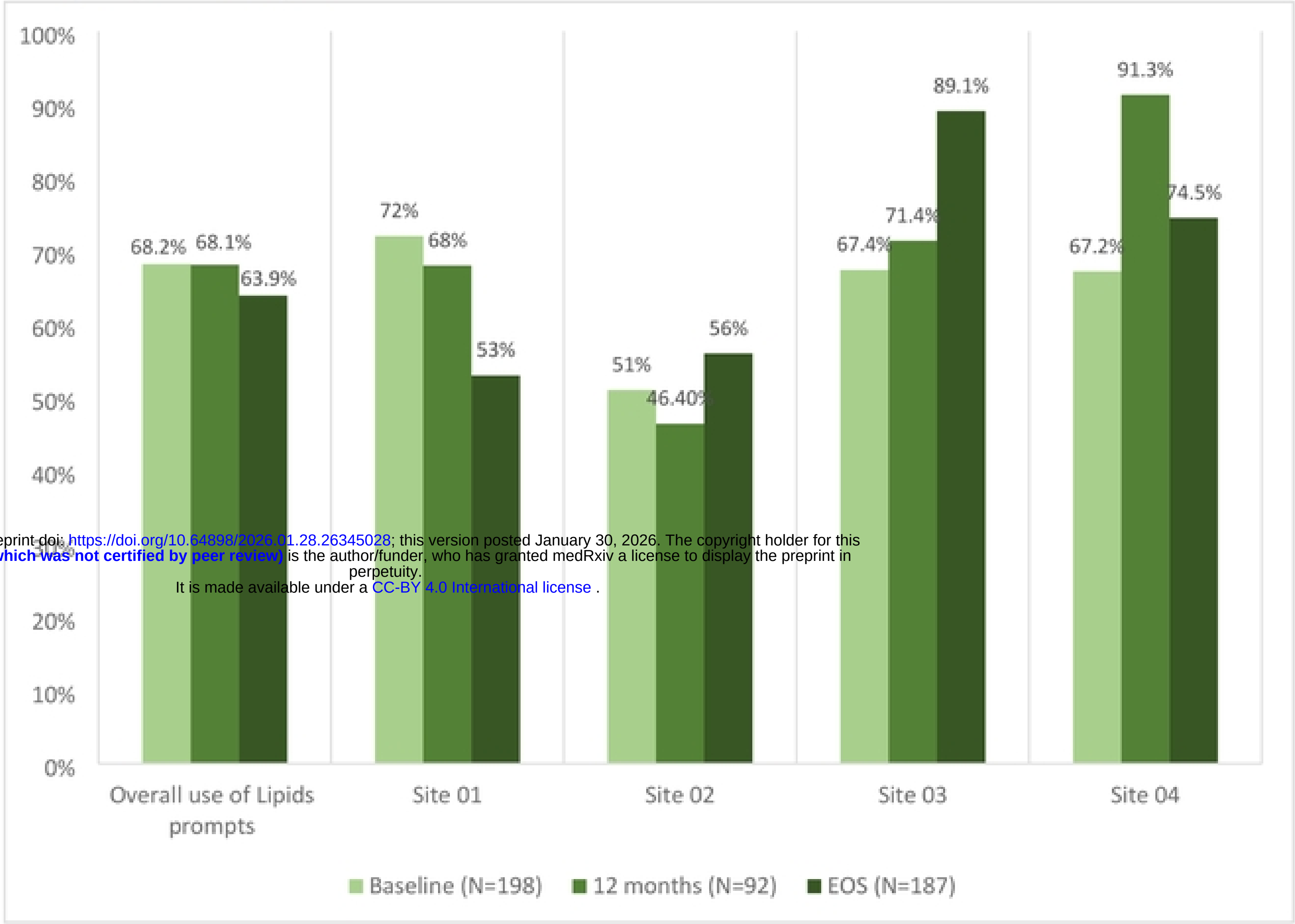

medRxiv preprint doi: https://doi.org/10.64898/2026.01.28.26345028; this version posted January 30, 2026. The copyright holder for this preprint (which was not certified by peer review) is the author/funder, who has granted medRxiv a license to display the preprint in perpetuity.
It is made available under a CC-BY 4.0 International license .

d) Glycemia eDSS Prompts

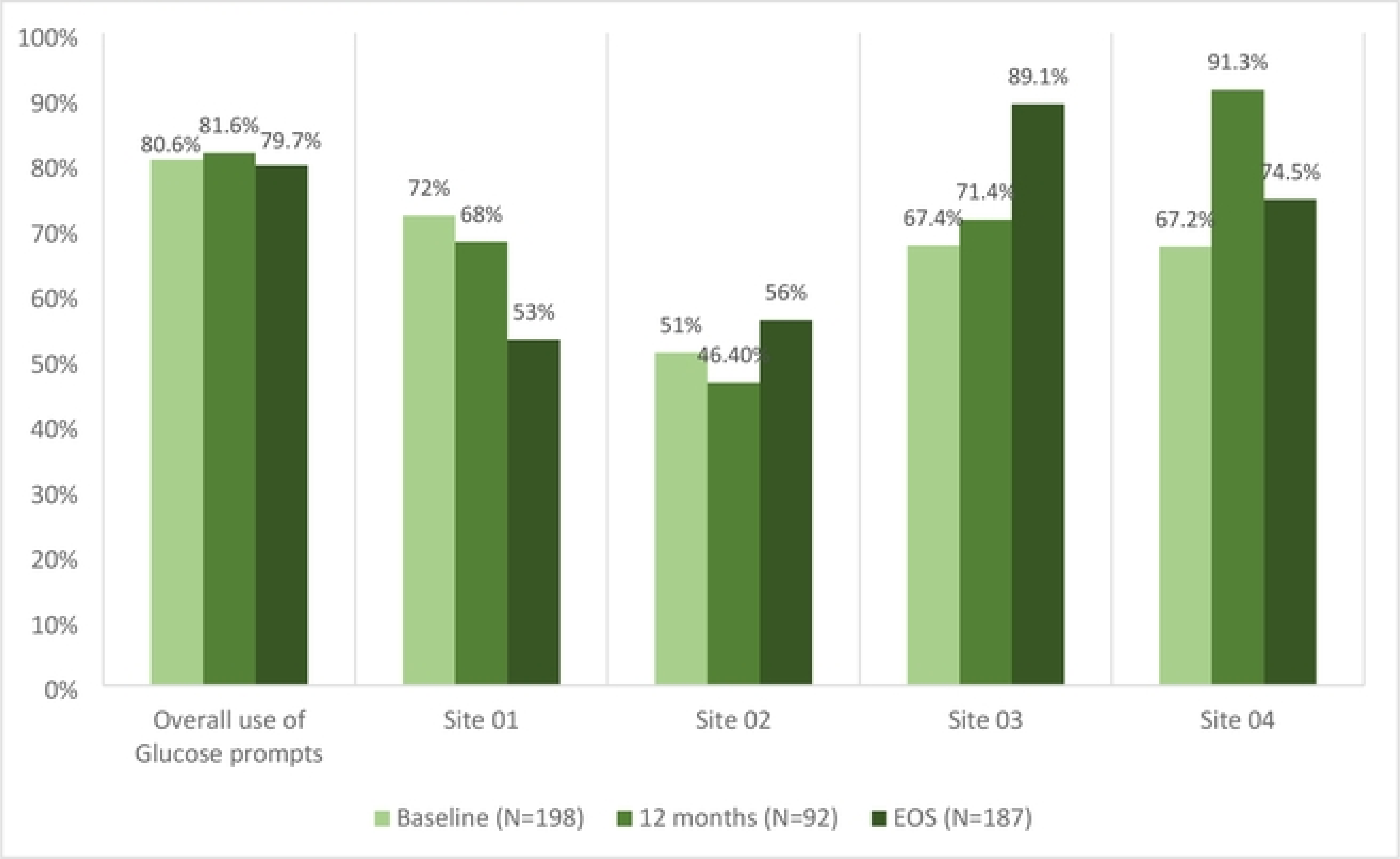

Table S.3.: Self-reported timeliness of care and type of reminders received

	Baseline		End of Study (EOS)		p-value*
	C-QIP strategy (N=198)	Usual care (N=203)	C-QIP strategy (N=187)	Usual care (N=192)	
**Total time spend for lab tests, include waiting (in minutes; Median, IQR)**	30 (15, 45)	20 (15, 35)	20 (15, 40)	30 (15, 60)	**<0.001**
**Total time spend waiting for consultation (in minutes; Median, IQR)**	20 (10, 40)	20 (10, 60)	15 (10, 25)	20 (10, 40)	0.13
**Total time spend in-person with doctor (in minutes; Median, IQR)**	9 (5, 10)	8 (5, 10)	10 (5, 10)	7 (5, 10)	**<0.001**
**Type of reminders received**					
Verbal reminder at current visit	147 (74.2%)	143 (70.4%)	130 (65.7%)	134 (66%)	0.95
Phone call for upcoming appointment	56 (28.3%)	71 (35%)	93 (47%)	57 (28.1%)	**<0.001**
Phone call if missed appointment	43 (21.7%)	34 (16.8%)	140 (70.7%)	9 (4.4%)	**<0.001**
Phone call to go for lab investigations	13 (6.6%)	9 (4.4%)	82 (41.4%)	1 (0.5%)	**<0.001**

**p-value is for the difference between the two groups at end of study*

medRxiv preprint doi: https://doi.org/10.64898/2026.01.28.26345028; this version posted January 30, 2026. The copyright holder for this preprint (which was not certified by peer review) is the author/funder, who has granted medRxiv a license to display the preprint in perpetuity.
It is made available under a CC-BY 4.0 International license.

Table S.4.: Self-reported family support at baseline and end of study.

Family has helped the participant to	Baseline		End of Study (EOS)		p-value*
	C-QIP strategy (N=198)	Usual care (N=203)	C-QIP strategy (N=198)	Usual care (N=203)	
Eat healthy	182 (91.9%)	146 (71.9%)	187 (94.4%)	138 (68%)	
Exercise	150 (75.8%)	126 (62.1%)	185 (93.4%)	132 (65%)	0.06
Quit smoking *(If said yes/quit to smoking)*	**N=35** 14 (40%)	**N=26** 7 (15.6%)	**N=45** 9 (34.6%)	**N=38** 1 (2.6%)	**<0.001**
**Family reminds participant to**					
Eat healthy	176 (88.9%)	141 (69.5%)	187 (94.4%)	138 (68%)	
Exercise	157 (79.3%)	129 (63.6%)	181 (91.4%)	127 (62.6%)	0.06
Quit smoking *(If said yes/quit to smoking)*	**N=35** 14 (40%)	**N=26** 8 (17.8%)	**N=45** 8 (30.8%)	**N=38** 1 (2.6%)	**<0.001**
**Family joins with the participant to**					
Eat healthy	170 (85.9%)	136 (67%)	186 (93.9%)	137 (67.5%)	0.39
Exercise	103 (52%)	75 (37%)	127 (64.1%)	96 (47.3%)	0.68
Quit smoking *(If said yes/quit to smoking)*	**N=35** 7 (20%)	**N=26** 2 (4.4%)	**N=45** 2 (7.7%)	**N=38** -	**0.05**

**p-value is for the difference between the two groups at EOS*

medRxiv preprint doi: https://doi.org/10.64898/2026.01.28.26345028; this version posted January 30, 2026. The copyright holder for this preprint (which was not certified by peer review) is the author/funder, who has granted medRxiv a license to display the preprint in perpetuity.
It is made available under a CC-BY 4.0 International license .

Figure S.2: Self-reported medication adherence at EOS by prespecified sub-groups

Subgroup	Relative Risk (95% CI)	P Value
Overall	1.08 (1.04–1.12)	0.001
Age (years)		0.204
<=49	1.03 (0.98–1.09)	
50-64	1.09 (1.03–1.16)	
>=65	1.10 (1.01–1.20)	
Sex		0.830
Male	1.07 (1.03–1.12)	
Female	1.08 (1.00–1.17)	
Education (years)		0.441
<5	0.99 (0.96–1.01)	
5-10	1.16 (1.07–1.25)	
10-12	1.10 (0.98–1.22)	
>12	1.01 (0.96–1.07)	
Household Income (INR)		0.270
<15000	1.00 (0.97–1.04)	
15000-30000	1.12 (1.05–1.19)	
>30000	1.08 (1.00–1.17)	
Facility type		0.001
Public	1.01 (0.98–1.04)	
Private	1.14 (1.07–1.22)	
BMI (kg/m²)		0.840
<25	1.11 (1.05–1.18)	
>=25	1.04 (1.00–1.09)	
Hypertension		0.803
No	1.08 (1.03–1.12)	
Yes	1.09 (1.02–1.16)	
Diabetes		0.143
No	1.05 (1.00–1.10)	
Yes	1.11 (1.04–1.19)	
Coronary heart disease		0.041
No	1.20 (1.06–1.36)	
Yes	1.04 (1.01–1.08)	
Heart failure		0.100
No	1.09 (1.04–1.14)	
Yes	1.02 (0.94–1.09)	
Stroke		0.001
No	1.02 (0.98–1.05)	
Yes	1.49 (1.26–1.77)	
Blood Pressure		0.140
<140/90 mmHg	1.06 (1.02–1.10)	
>=140/90 mmHg	1.16 (1.04–1.28)	
LDL-cholesterol		0.243
<100 mg/dl	1.06 (1.01–1.11)	
>=100 mg/dl	1.13 (1.03–1.24)	

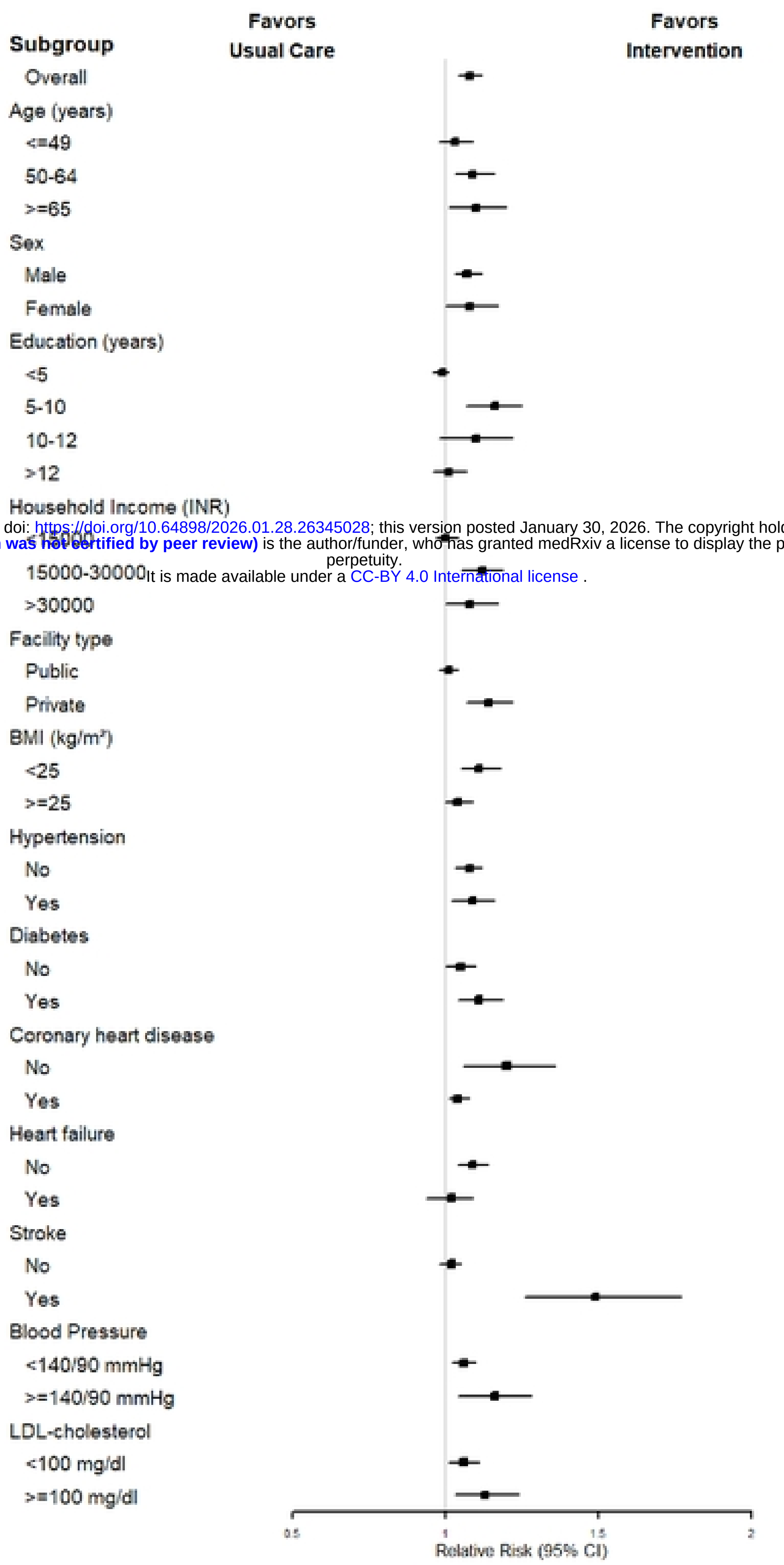

medRxiv preprint doi: https://doi.org/10.64898/2026.01.28.26345028; this version posted January 30, 2026. The copyright holder for this preprint (which was not certified by peer review) is the author/funder, who has granted medRxiv a license to display the preprint in perpetuity.
It is made available under a CC-BY 4.0 International license .

CI=confidence interval; BMI=body mass index (Kg/m²); LDLc=low-density lipoprotein cholesterol, mg/dl=milligrams per deciliter; mmHg=millimoles per mercury; INR=Indian rupees

Figure S.3: Mean systolic blood pressure reduction at end-of-study by prespecified subgroups

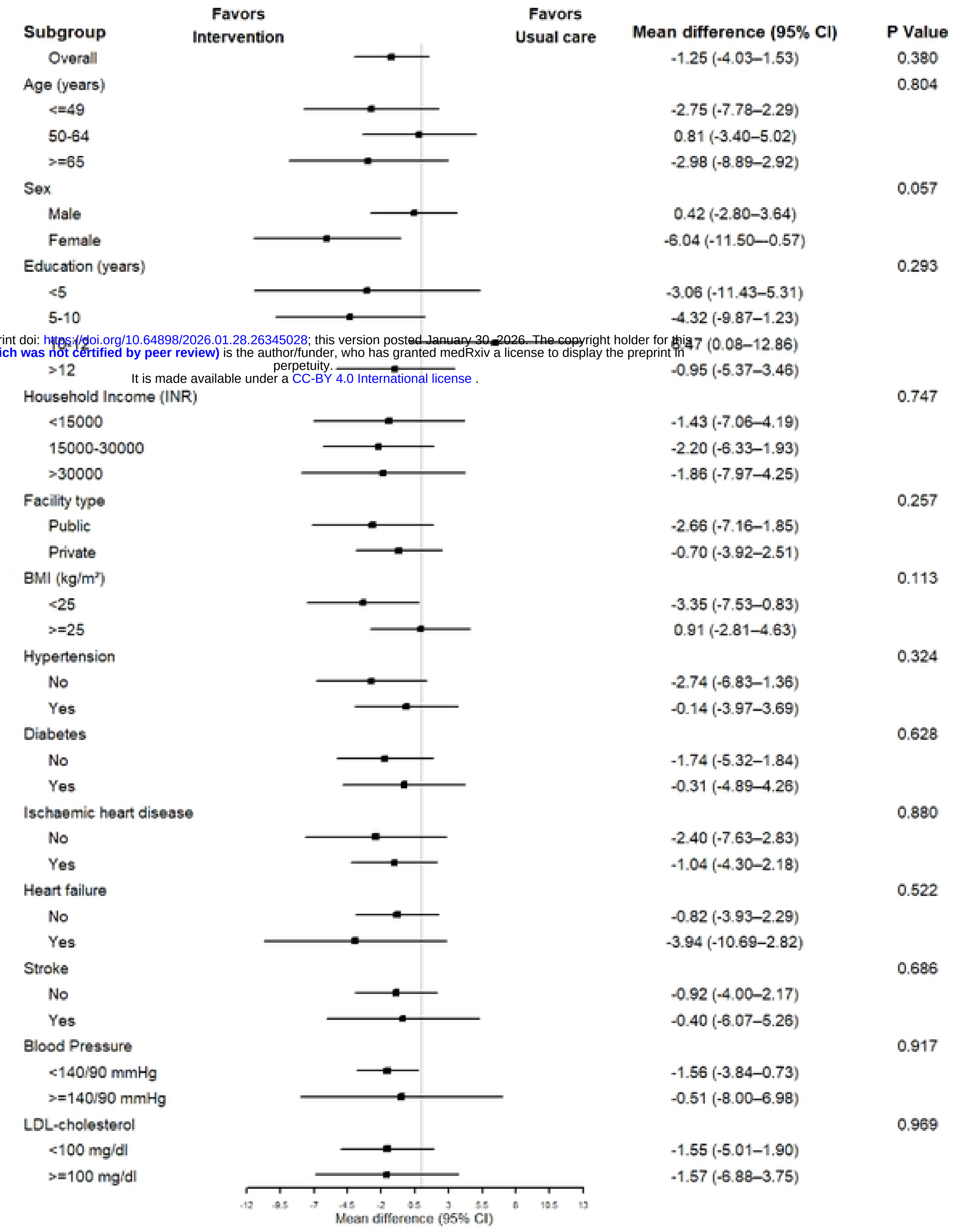

medRxiv preprint doi: https://doi.org/10.64898/2026.01.28.26345028; this version posted January 30, 2026. The copyright holder for this preprint (which was not certified by peer review) is the author/funder, who has granted medRxiv a license to display the preprint in perpetuity.
It is made available under a CC-BY 4.0 International license .

CI=confidence interval; BMI=body mass index (Kg/m²); LDLc=low-density lipoprotein cholesterol, mg/dl=milligrams per deciliter; mmHg=millimoles per mercury

Table S.5.: Serious adverse events

Event description	Total events reported in the trial		
	**Total**	**C-QIP strategy**	**Usual care**
**Deaths (all cause)**	**19**	**9**	**10**
Cardiovascular deaths	11	6	5
Other deaths*	8	3	5
Death followed by severe illness			3
Road traffic accident			1
Cancer complications		1	
Deteriorating health		1	
Post-operation complications		1	
Difficulty in breathing			1
**Hospitalizations**	**5**	**5**	**0**
Angina on exertion	2	2	
Chest pain	1	1	
Anteroseptal Myocardial Infarction	1	1	
Planned angiography	1	1	

medRxiv preprint doi: https://doi.org/10.64898/2026.01.28.26345028; this version posted January 30, 2026. The copyright holder for this preprint (which was not certified by peer review) is the author/funder, who has granted medRxiv a license to display the preprint in perpetuity.
It is made available under a CC-BY 4.0 International license .

Table S.6.: Comparison of baseline characteristics of participants who completed the EOS visit versus participants who did not complete EOS visit

	Completed EOS visit	Lost to follow up	p-value
	N=379	N=22	
Age (years; mean, SD)	57.1 (11.7)	62.7 (11.0)	0.03
Male, n (%)	277 (73.1%)	16 (72.7%)	0.97
Education, (years; mean, SD)	10.7 (5.6)	11.6 (4.3)	0.44
Household Income (monthly) Indian rupees, median (IQR)	20000 (10000, 30000)	27500 (20000, 30000)	0.05
Systolic BP (mm Hg; mean, SD)	127.4 (18.1)	134.5 (19.9)	0.07
Diastolic BP (mm Hg; mean, SD)	76.2 (11.4)	78.8 (14.3)	0.30
Total cholesterol (mg/dL; mean, SD)	144.3 (44.2) (n=370)	149.0 (37.7) (n=22)	0.63
LDLc (mg/dL; mean, SD)	80.2 (37.4) (n=367)	84.7 (35.2) (n=21)	0.59
FBG (mg/dL; mean, SD)	123.0 (55.2) (n=182)	87.6 (23.6) (n=4)	0.20
HbA1c (%; mean, SD)	6.7 (1.6) (n=225)	8.0 (1.7) (n=7)	0.03
**Medical history**			
Coronary heart disease, n (%)	286 (75.5%)	10 (45.5%)	0.002
Heart failure, n (%)	76 (20.1%)	6 (27.3%)	0.41
Ischemic stroke, n (%)	65 (17.2%)	9 (40.9%)	0.005
Hypertension, n (%)	178 (47.0%)	15 (68.2%)	0.05
Diabetes, n (%)	155 (40.9%)	13 (59.1%)	0.09
**Medications use**			
Antiplatelet	336 (88.7%)	15 (68.2%)	0.005
Lipid lowering (Statin)	330 (87.1%)	14 (63.6%)	0.002
ACEi/ARB	227 (59.9%)	12 (54.5%)	0.62
ARNI (n=82; heart failure patients)	24 (32%)	1 (17%)	0.44
Beta-blockers	269 (71.0%)	15 (68.2%)	0.78
Calcium Channel Blockers	64 (16.9%)	7 (31.8%)	0.07
Diuretics	29 (7.7%)	1 (4.5%)	0.59
Did participant forget to take medications (in last 1 month)?	56 (14.8%)	3 (13.6%)	0.96
On feeling worse does the participant stops medication on his/her own?	19 (5.0%)	0 (0.0%)	0.28

*EOS=End of study visit; SD=standard deviation, mg/dl=milligrams per deciliter, BP=blood pressure

medRxiv preprint doi: https://doi.org/10.64898/2026.01.28.26345028; this version posted January 30, 2026. The copyright holder for this preprint (which was not certified by peer review) is the author/funder, who has granted medRxiv a license to display the preprint in perpetuity.
It is made available under a CC-BY 4.0 International license .
